# Broadband Electromagnetic Absorption Effect of Topological Structure Using Carbon Nanotube Based Hybrid Material

**DOI:** 10.3390/ma15144983

**Published:** 2022-07-18

**Authors:** Ying Zhang, Qing Shen, Yixing Huang, Qin Lu, Jijun Yu

**Affiliations:** 1 China Academy of Aerospace Aerodynamics, Beijing 100074, China; zhangying7010102@126.com (Y.Z.); qshen2@sina.com (Q.S.); luqin@tsinghua.org.cn (Q.L.); 2 Institute of Advanced Structure Technology, Beijing Institute of Technology, Beijing 100081, China

**Keywords:** microwave absorbing, topological structure, fractal dimension, carbonyl iron, 3D printing

## Abstract

The development of microwave absorbing technology raises the demands for all-band absorption. The topological structures expand the frequency range of electromagnetic wave absorption and eliminate the differences caused by scattering in different incident directions. The multi-wall carbon nanotube and carbonyl iron particles were mixed with polylactic polymer to fabricate filaments for fused deposition. The distribution characteristics of the structures using carbonyl iron/carbon nanotube hybrid material for the key absorption frequency band are obtained. The reflectivity of the honeycomb structure in X and Ku bands is verified experimentally through the preparation method of fused deposition modeling 3D printing. With the decrease of the fractal dimension number, the electromagnetic loss performance basically increases. Preliminary research results showed that the topological structure could significantly expand the absorbing frequency range, and the effective frequency band less than −10 dB is 2–40 GHz, which has a clear application potential for radar absorption.

## 1. Introduction

Microwave absorbing materials are important for the radar stealth technology, and can be used for coating, patch and structural material [1,2]. In the civil fields, such as microwave radiation body protection and electromagnetic compatibility, the materials are also used to varying degrees [3,4]. The electromagnetic properties of traditional absorbing composites, such as ferrites, magnetic metals, and carbon-based materials, are determined by the properties of the microscopic particles that make up the materials. In recent years, researchers have improved the dielectric loss performance of absorbing materials by introducing carbon nanotubes, graphene, pyrolytic graphite and other electrical loss nano materials into resin matrix materials, and increased the magnetic loss performance of the materials by introducing nano-ferrite, nano-alloy, nano-metal oxide and other magnetic particles [5,6,7,8]. The composite electromagnetic loss performance can also be improved by mixing electric loss and magnetic loss nanoparticles in a certain proportion. However, traditional absorbing composite materials are mainly used in X and Ku bands, which are commonly used to absorb radar waves, and are not suitable for all-band absorption.

To extend the frequency range, broadband absorption of absorbing materials can be effectively realized through the design of structural units of in-plane metamaterial. In 2008, Landy et al. first designed a three-layer metal surface resonance structure based on metamaterials, and selected the corresponding medium to achieve perfect wave absorption at the single-frequency resonance point [9]. Since then, absorbers with fixed narrow bandwidths based on metallic structures have been extensively studied [10,11,12]. Wang et al. designed an absorber with dual-frequency resonant point by utilizing the coupling effect of the double-ring metal structure, and increased the absorption bandwidth at the high-frequency resonant point by changing the symmetry of the double-ring structure [13]. Li et al. used a hexagonal frequency-selective surface periodic structure with high dielectric constant to realize the three-frequency absorption effect of the absorber [14]. In 2015, Agarwal et al. increased the absorption bandwidth to a certain extent based on a study of non-traditional tapered helical absorbers [15]. The above studies improved the absorption bandwidth of the absorber by arranging multiple resonators of different sizes in a single cell or by stacking multilayer resonators and dielectric layers of different thicknesses. Although the above method can have good absorbing performance at the resonance point, it is usually only able to absorb narrow-band waves at a single or multiple resonance points, and cannot achieve broadband absorbing effects.

To better accommodate the functions of broadband absorbing/mechanical load bearing, researchers integrate the functions into one structure by means of integrated design of loss metastructure and composite panel [16,17,18]. In 2018, Huang fabricated an electromagnetic dual-loss nanopaste of epoxy resin and silicone rubber into periodic subwavelength structures. Considering the overall impedance matching, dual mechanical reinforcement of carbon fiber bottom plate and glass fiber panel is carried out to obtain a dual-functional periodic metastructure with both mechanical load bearing performance and electromagnetic wave absorbing performance [19,20]. 

In this paper, a topological absorbing structure is proposed to realize broadband absorbing. The topological structure is a kind of mechanical load bearing structure, which can also solve the problem of compatibility of broadband absorption and mechanical load bearing, and is easy to be applied in engineering practice. The fractal dimension number of the topological structure can characterize the complexity of the geometrical features of the structure, and the geometrical features of the structure affect the overall absorbing performance of the material. This paper gives the wave absorption and fractal dimension characteristics of the topology structure and focuses on the analysis of the broadband absorption characteristics of the topological structure in the vertical incident direction of electromagnetic waves.

## 2. Materials and Methods

### 2.1. Material and Characterization

The carbonyl iron (Shaanxi Xinghua Chemical Group Co., Ltd., Shaanxi, China, average diameter of 3.2 μm)/multi-wall carbon nanotube (Shenzhen Nano Port Co., Ltd., Shenzhen, China with average length and diameter of 2 μm and 60 nm)/polylactic polymer (CI/MWCNT/PLA) hybrid material was selected as the wave absorbing material. Carbonyl iron particles were used to increase complex permeability of the composite. To prepare the CI/MWCNT/PLA hybrid material, the carbonyl iron, carbon nanotube and polylactic polymer powder were firstly dried in drying oven at 80 °C for 12 h. Then, the mixture with the volume ration of CI/MWCNT/PLA being 50:2:48 was dispersed in planetary mixer at 60 r/min for 8 h. The hybrid powder was melted, mixed and extruded through the granulator to prepare hybrid material particles. Finally, the hybrid material particles were poured into the single-screw extruder to extrude 1.75 ± 0.05 mm hybrid material filaments under the action of heat melting and screw friction shear. The filaments were printed by fused deposition to designed structure form. The status of carbonyl iron particles in the fabricated hybrid composite was solid as no chemical reaction occurred for the iron in the solidification process. The density of the fabricated composite was 3.1 g/cm^3^.

**Figure 1:**
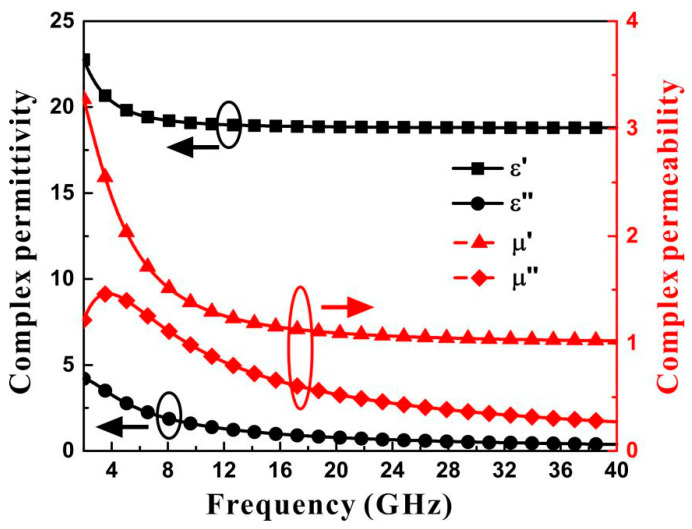
Experimental complex permittivity and complex permeability of CI/MWCNT/PLA hybrid material at 2–40 GHz.

The complex permittivity and complex permeability of hybrid material were measured by the waveguide method in the frequency band of 2–40 GHz. The reflectivity was measured by arch frame far-field method. Figure 1 shows the measured electromagnetic parameters of CI/MWCNT/PLA hybrid material after multi-order Debye model fitting. 

**Figure 2:**
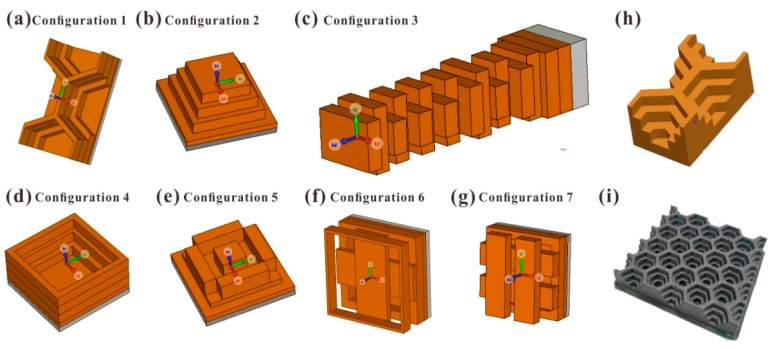
(**a**–**g**) Seven electromagnetic metastructure configurations (Configuration 1 to 7) for fractal dimension number calculation and reflectivity simulation. (**h**) Designed gradient honeycomb cell for 3D printing. (**i**) The printed gradient honeycomb metastructure sample fabricated by fused filament deposition with blue print shown in (**h**).

The mechanical lattice structure with fractal dimension is selected to analyze the wave absorption characteristics of the all-band. The base material of the lattice structure is made of absorbing materials. The optimal configuration with optimization potential is selected, which can maintain the broadband absorbing performance. The seven metastructure unit cell configurations for fractal dimension number calculation and reflectivity simulation are shown in Figure 2a–g. The designed gradient honeycomb cell for 3D printing and the printed array sample with fused filament deposition are shown in Figure 2h,i respectively.

### 2.2. Simulation Method and Analysis Model

The finite element method can be used to solve the differential Maxwell’s equations under given boundary conditions [21]. According to the constitutive relation of medium in electromagnetism, the vector wave equation can be derived from Maxwell’s equations.

(1) $$\nabla \times \left(\mu_{r}^{-1}\nabla \times E\right)-k_{0}^{2}\varepsilon_{r}E=-j\omega \mu_{0}J$$

where $\varepsilon_{r}$ and $\mu_{r}$ are the relative permittivity and relative permeability of the medium, respectively, $\mu_{0}$ is the permeability in vacuum, $k_{0}$ is the wavenumber in vacuum, ω is the operating angular frequency, and J is the excitation current density.

When calculating the microwave absorbing performance of the honeycomb structure, the size of the unit cell of the structure is drawn up. The unit cell analysis model is established, and the electromagnetic periodic boundary condition is applied. The complex permittivity, complex permeability, electrical conductivity, and sheet resistance, which characterize the electromagnetic parameters of the absorbing material, can be expressed as follows

(2) $$\varepsilon_{nc}={\varepsilon^{\prime }}_{n}-j\left(\frac{\sigma }{\omega }+{\varepsilon^{\prime \prime }}_{n}\right)$$

(3) $$\mu_{nc}={\mu^{\prime }}_{n}-j{\mu^{\prime \prime }}_{n}$$

(4) $$R_{s}=\frac{1}{\sigma d}$$

where ${\varepsilon^{\prime }}_{n}$ is the real part of the complex permittivity, ${\mu^{\prime }}_{n}$ is the real part of the complex permeability, σ is the conductivity, ω is the angular frequency of the electromagnetic wave, ${\varepsilon^{\prime \prime }}_{n}$ is the imaginary part of the complex permittivity, ${\mu^{\prime \prime }}_{n}$ is the imaginary part of the complex permeability, $R_{s}$ is the square impedance of the resistive film, $\varepsilon_{nc}$ is the complex permittivity, $\mu_{nc}$ is the complex permeability, and d is the film thickness. Together with $d_{n}$ thickness, there are six variables for each medium. Then the electromagnetic field and reflectivity are calculated by the finite element method in the frequency domain in CST.

### 2.3. Calculation of Fractal Dimension Number

The fractal dimension of the multi-scale structure is calculated by the calculation method of Hausdorff dimension [22]. For a geometric object with measure volume *V*, the geometric object can be filled by spheres with a radius of *r*, then the required number of spheres should be

(5) $$N_{r}=\frac{V}{\frac{4}{3}\pi r^{3}}\propto 1/r^{3}$$

Extending to the general situation, for a geometric object with volume *A* and fractal dimension $D_{f}$, the number of spheres required to measure with radius *r* is:

(6) $$N_{r}=A/Cr^{D_{f}}\propto 1/r^{D_{f}}$$

Here *C* is the structure factor. Thus, the fractal dimension is

(7) $$D_{f}=\frac{\ln N_{r}}{\ln\left(1/r\right)}$$

The calculation of the fractal dimension number of the configuration in this paper includes three main steps: (1) use the cube grids of different scales to contain the three-dimensional space occupied by the configuration; (2) calculate the number of cube grids containing objects; (3) perform the least squares regression analysis on $\ln N_{r}$ and ln(1/r), and the slope of the fitted line is the fractal dimension number. The fractal dimension statistical model and profile grid of the configuration are used. According to the geometric characteristics of the configuration, the characteristic profile of the configuration is cut off, the number of grids occupied by the characteristic profile is counted, and then the number of cubic grids occupied by the overall configuration is counted.

## 3. Results and Discussion

### 3.1. Experimental Verification

**Figure 3:**
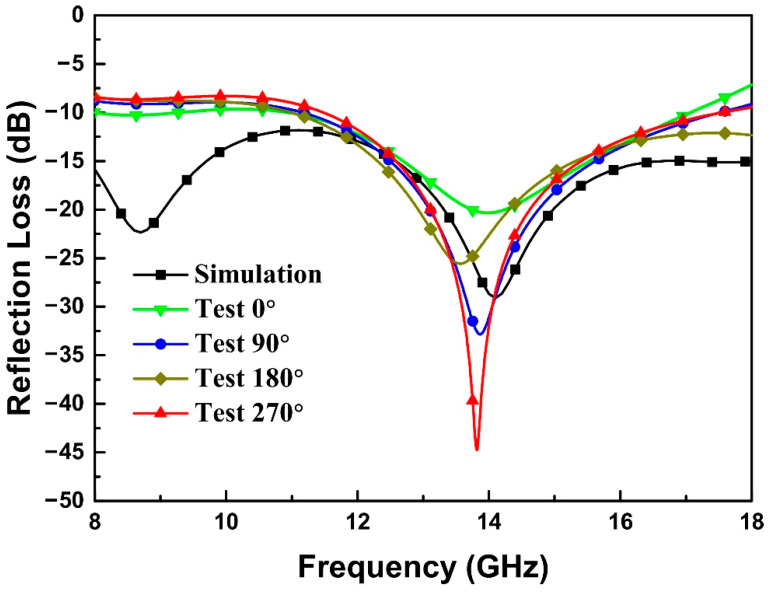
Normal experimental reflectivity of gradient honeycomb metastructure shown in Figure 2h, i in X and Ku bands from four directions in comparison with the simulated result.

To verify the simulation method and analysis model of the topological structure, the honeycomb configuration is selected as the structural unit cell considering the characteristics of the fused deposition modeling 3D printing process. The honeycomb is a basic and practical structure form in massive manufacturing. Moreover, the design results indicate that the honeycomb had broadband absorption performance with suitable design. The unit cell size of the honeycomb structure is shown in Figure 2h, the thickness of the structure is 11 mm, and the printed object is shown in Figure 2i. The side length of the hollow hexagon from the bottom layer to the top layer is 4.2 mm, 6.5 mm and 8.4 mm. The thickness of each layer from the bottom to the top is 2.5 mm, 2.7 mm, 2.8 mm and 3 mm. The difference between the experimental and simulated reflectivity of −10 dB effective bandwidth in the frequency band of 8–18 GHz is shown in Figure 3. The simulation calculation results are consistent with the experimental results, indicating that the simulation calculation method is effective, and can be used to simulate the reflectivity calculation of multi-scale structures.

### 3.2. Reflectivity Calculation and Analysis

**Figure 4:**
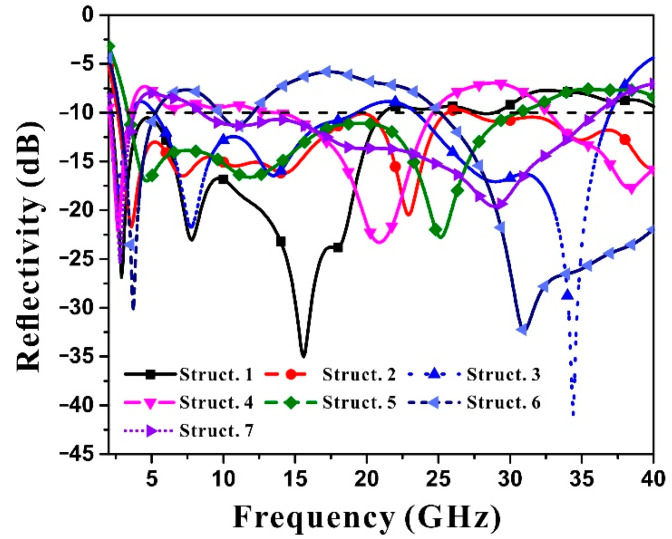
Simulated reflectivity of seven configurations at 2–40 GHz based on the measured electromagnetic parameters of carbonyl iron/carbon nanotube hybrid materials.

The simulated reflectivity of the seven topological structures in 2–40 GHz band is shown in Figure 4. Different configurations have different distribution characteristics for key absorption bands. Some configurations are focused on high-low frequency band coordination and mid-frequency band reduction, while other configurations are focused on low-frequency band absorption, and the rest configurations are focused on balanced distribution across the entire frequency range.

It can be found that the gradient honeycomb of Configuration 1 has strong electromagnetic wave absorption in the frequency band of 2–18 GHz, and has absorption peaks at 7.5 GHz and 15 GHz. Configuration 1 honeycomb (Figure 2a) has stronger absorbing capacity than the other 6 configurations at 2–18 GHz. However, its absorbing performance remains above −10 dB at 20–40 GHz, indicating that its absorption of high frequency band is inferior to that of the other 6 configurations. This is because the absorption of electromagnetic energy by the configuration causes a distribution difference between high frequency and low frequency, and the design of the size makes the electromagnetic energy absorption concentrated in the lower frequency band.

In contrast to Configuration 1, Configuration 3, the wood stack structure (Figure 2c), has a more even distribution in terms of electromagnetic absorption performance at 2–20 GHz and 22.5–37 GHz, balancing the electromagnetic energy loss effect in both low and high frequency bands, but its absorption depth in the low frequency band is inferior to that of Configuration 1, the gradient honeycomb. This shows that different configurations have important influences on the distribution of electromagnetic absorption frequency band. The wood stack configuration is more complex than the honeycomb configuration.

The complexity of Configuration 6 (Figure 2f) is higher than that of Configuration 1 honeycomb, but lower than that of Configuration 3 wood stack. However, its absorbing frequency band is evenly distributed throughout the whole frequency band, and has good electromagnetic absorption capacity in 2–12.5 GHz and 26–40 GHz bands, but it shows weakened absorption performance in the middle frequency band.

In addition, Configuration 2 ladder cones (Figure 2b), Configuration 4 (d), Configuration 5 (e), and Configuration 7 (g) all have the reflectivity distribution in full frequency band. However, in some frequency bands, the reflectivity is slightly higher than −10 dB, but the overall electromagnetic energy loss is relatively balanced. The four configurations are similar in complexity.

Since the seven lattice structures use the same electromagnetic double-loss material, the influence of material performance on the comprehensive broadband electromagnetic absorption performance can be ignored. The complexity of the configuration is closely related to the dispersion characteristics of the electromagnetic absorption. 

For the analysis of layers, Configurations 1, 2, 4, and 7 are 4-layer structure, Configuration 3 is a 16-layer structure, Configuration 5 is a 3-layer structure, and Configuration 6 is a 5-layer structure. The more layers there are, the more complex the structure is, and the electromagnetic absorption property of Configurations 3 and 6 are better than that of other configurations. 

The number of the configuration layers can show the qualitative relationship between configuration complexity and electromagnetic absorption performance to a certain extent. However, the increase in the number of layers cannot explain the strong absorption of Configuration 1 in low frequency band and the balanced absorption in high frequency band. Therefore, it is necessary to quantify the complexity of configuration and study the relationship between configuration and reflectivity according to the quantitative index of reflectivity dispersion characteristics.

### 3.3. Fractal Dimension Numbers of Seven Typical Configurations

The fractal dimensions of the seven configurations were calculated by fitting the slope of the straight lines linked by three points of ln(*N_r_*) and ln(1/*r*) pairs generated with different scales of grid cubes. Cubic grids with side lengths of 0.2 mm, 0.05 mm and 0.002 mm were selected to fill the three-dimensional space occupied by configurations, and the number of grids was counted. Configuration 1 is a 4-layer structure with a fractal dimension of 2.9695, Configuration 3 is a 15-layer structure with a fractal dimension of 2.8875, and Configuration 6 is a 5-layer structure with a fractal dimension of 2.9323. With the increase of configuration layers, the fractal dimension number decreases. A comparison of all the 4-layer configurations show that the difference in fractal dimensions of configurations is caused by the shapes and sizes of the structures. The fractal dimension numbers of the seven typical configurations are shown in Table 1. It can be seen that the fractal dimension can characterize the complexity of the geometric features of the configuration.

### 3.4. Correlation between Fractal Dimension and Electromagnetic Loss

**Figure 5:**
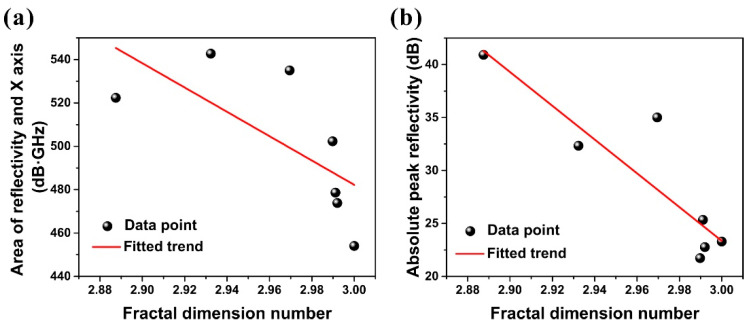
Quantitative correlation and fitted trends between the fractal dimension number of proposed structure and (**a**) area surrounded by reflectivity curve and *X*-axis, (**b**) peak value of reflectivity curve.

The fractal dimension number of the configuration can quantitatively characterize the complexity of the geometric features of the configuration, and is determined by the number of layers, shape and size of the configuration. Figure 5a,b show the quantitative relationship between fractal dimension number of the seven configurations and the area surrounded by the reflectivity and the *X*-axis (the line of y = 0) as well as the reflectivity peak. The referred configuration number is shown besides the data points. The reflectivity of Configurations 1, 3 and 6 has a larger area enclosed by the *X*-axis and a higher reflectivity peak. Therefore, the configuration with smaller fractal dimension has better electromagnetic loss performance. From the point of view of design, the configuration with higher complexity has more structural size parameters, so the optimal configuration design can be selected to meet the impedance matching characteristics and attenuation characteristics of the structure for electromagnetic wave. Therefore, fractal dimension number, as a quantitative index to measure the complexity of structures, can be used to select the optimal configuration design schemes.

## 4. Conclusions

In this study, seven typical lattice structures with fractal dimension characteristics were selected to analyze the characteristics of wave absorption and fractal dimension of the carbonyl iron/carbon nanotube hybrid material. The reflectivity of the honeycomb structure with fractal dimensional characteristics in X and Ku bands was verified experimentally. The simulation results were consistent with the experimental results, which shows the effectiveness of the method. The seven typical fractal structures can basically cover the −10 dB effective bandwidth of 2–40 GHz for the vertical incident direction of electromagnetic waves. Different configurations have different distribution characteristics for key absorption frequency bands. There is a clear correlation between the fractal dimension and the electromagnetic loss performance. With the decrease of fractal dimension from 3.0000 to 2.8875, the electromagnetic loss performance increases monotonically.

## Figures and Tables

**Table 1:** Fractal dimension number of the seven configurations shown in Figure 2.

Configuration	1	2	3	4	5	6	7
Fractal dimension number	2.9695	2.9898	2.8875	3.0000	2.9920	2.9323	2.9911

## Data Availability

The data presented in this study are available on request from the corresponding author.
